# Complete mitochondrial genome of a rare diatom (Bacillariophyta) *Proschkinia* and its phylogenetic and taxonomic implications

**DOI:** 10.1080/23802359.2018.1535852

**Published:** 2018-11-25

**Authors:** Romain Gastineau, So-Yeon Kim, Claude Lemieux, Monique Turmel, Andrzej Witkowski, Jong-Gyu Park, Byoung-Seok Kim, David G. Mann, Edward C. Theriot

**Affiliations:** a Palaeoceanology Unit, Faculty of Geosciences, and Natural Sciences Research and Educational Centre, University of Szczecin, Szczecin, Poland;; b Department of Oceanography, College of Ocean Science & Technology, Kunsan National University, Republic of Korea;; c Département de biochimie, de microbiologie et de bio-informatique, Institut de Biologie Intégrative et des Systèmes, Université Laval, Québec, Québec, Canada;; d Faculty of Marine Applied Biosciences, College of Ocean Science & Technology, Kunsan National University, Gunsan, Republic of Korea;; e Royal Botanic Garden Edinburgh, Edinburgh, Scotland, UK;; f Institute of Agriculture and Food Research and Technology (IRTA), Sant Carles de la Ràpita, Spain;; g Department of Integrative Biology, University of Texas at Austin, Austin, TX, USA

**Keywords:** Diatoms, fistula, multigene phylogeny, mitogenome, Proschkinia

## Abstract

We obtained the complete mitogenome of *Proschkinia* sp. strain SZCZR1824, a strain belonging to a poorly known diatom genus with no previous molecular data. This genome is 48,863 bp long, with two group I introns in *rnl* and three group II introns in *cox*1. Using mitogenomic data, *Proschkinia* sp. was recovered with *Fistulifera solaris*, far distant from *Navicula* and *Nitzschia*, two genera with which *Proschkinia* has sometimes been associated based on morphology.

*Proschkinia* is a rare genus of diatom of controversial higher classification. Originally classified as a relative of *Nitzschia* on the basis of light microscopy (Karayeva 1978), *Proschkinia* is now classified within its own family, Proschkiniaceae (Round et al. 1990), within the Naviculales, without any specific hypothesis as to its relationship with other Naviculales. Additional SEM studies failed to further resolve you need the relationship of *Proschkinia* (Brogan and Rosowski 1988; Cox 1988, 2012).

We sequenced the complete mitochondrial genome of *Proschkinia* sp. strain SZCZR1824, a strain displaying similarities with *Proschkinia complanatoides*, and created a comparative data set from this and published diatom mitochondrial genes in order to better resolve the phylogenetic position of *Proschkinia*.

*Proschkinia* sp. strain SZCZR1824, originating from Padori Beach on the Yellow Sea coast of Korea (36°44′15.0″N, 126°07′49.7″E) was obtained from Kunsan National University (Korea). Total DNA was extracted following Doyle and Doyle (1990). Paired-end sequencing (150 bp) was conducted by the Beijing Genomic Institute (Shenzhen) on HiSeq 4000, with inserts of 300 bp, for a total of ca. 30 million reads. Assembly was performed using Ray 2.3.1 (Boisvert et al. 2010) with a k-mer of 35. Gene identification was done using custom tools developed at Laval University (Gagnon 2004).

A permanent slide with cleaned frustules of SZCZR1824 is kept in the collection of the University of Szczecin. Frozen DNA and pellets of cells are also being stored in Szczecin at −20 °C.

The mitogenome of *Proschkinia* sp. SZCZR1824 (MH800316) is 48,863 bp long and encodes two rRNAs, 22 tRNAs and 33 proteins, for a total of 57 gene products. In addition, there are two free-standing open reading frames (orf143 and orf243) with no obvious function. The large subunit rRNA gene (*rnl*) is interrupted by two group I introns, whereas *cox1* is interrupted by three large group II introns. Each of the *cox1* introns contains a putative reverse transcriptase gene (orf714, orf789 and orf1002) and BlastP searches using these gene products as queries identified putative diatom proteins encoded by *cox1* introns: YP_009495514 (*Psammoneis japonica*) for orf714, YP_009144752 (*Pseudo-nitzschia multiseries*) for orf789 and AVR57660 (*Halamphora* sp.) for orf1002.

A maximum-likelihood phylogenetic analysis was performed on a concatenated data set of *cox1*, *cox2*, *cox3*, *cob*, *nad2*, *nad4*, *nad5*, and *nad11* from 16 diatoms using RAxML 8.2.12 (Stamatakis 2014). *Proschkinia* sp. was recovered as sister to *Fistulifera solaris* (Figure 1), which was previously placed in the Stauroneidaceae (Cox 2015) or Naviculaceae (NCBI taxonomy, accessed 25 August 2018) in the Naviculales. These two species formed a larger, strongly supported clade with *Berkeleya fennica* (Berkeleyales) and *Didymosphenia geminata* (Cymbellales). Bacillariales species (*Nitzschia*, *Pseudo-nitzschia* and *Cylindrotheca*) and *Navicula ramosissima* are separated by several nodes from *Proschkinia*, a result incongruent with the taxonomic placements reported for the latter diatom by Karayeva (1978) and Round et al. (1990). *Fistulifera*, like *Proschkinia*, possesses a special structure, called a ‘fistula’, between the raphe slots at the valve center (Lange-Bertalot 1997; Zgrundo et al. 2013). Based on this shared feature and the mitochondrial phylogeny presented here, we propose that *Fistulifera* and *Proschkinia* belong to the same family, Proschkiniaceae.

**Figure 1.**
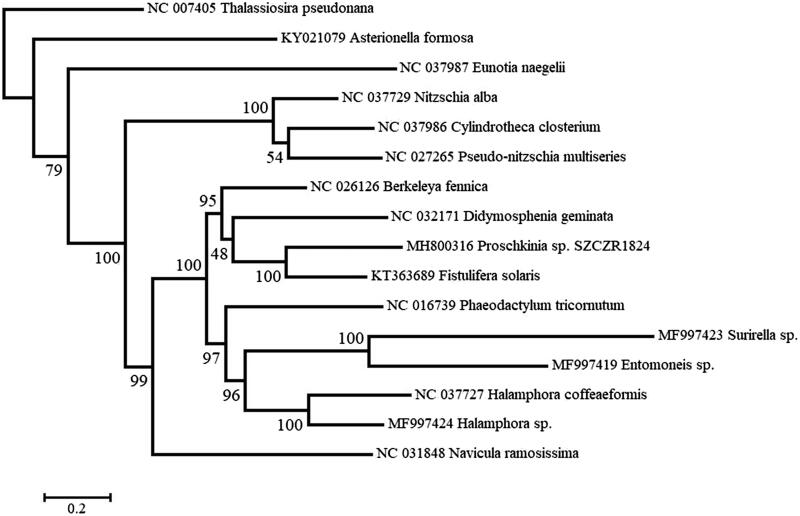
Maximum likelihood phylogeny obtained on concatenated mitochondrial genes (*cox1*, *cox2*, *cox3*, *cob*, *nad2*, *nad4*, *nad5*, and *nad11*) of *Proschkinia* sp. and other diatoms, with *Thalassiosira pseudonana* being the outgroup. Numbers next to nodes are support values obtained after 100 bootstrap replicates.
